# Estimation of health impact from digitalizing last-mile Logistics Management Information Systems (LMIS) in Ethiopia, Tanzania, and Mozambique: A Lives Saved Tool (LiST) model analysis

**DOI:** 10.1371/journal.pone.0258354

**Published:** 2021-10-25

**Authors:** Jenna Fritz, Tara Herrick, Sarah Skye Gilbert

**Affiliations:** 1 Market Dynamics, PATH, Seattle, Washington, United States of America; 2 Digital Square, PATH, Seattle, Washington, United States of America; University of Washington, UNITED STATES

## Abstract

**Background:**

Digital health has become a widely recognized approach to addressing a range of health needs, including advancing universal health coverage and achieving the Sustainable Development Goals. At present there is limited evidence on the impact of digital interventions on health outcomes. A growing body of peer-reviewed evidence on digitalizing last-mile electronic logistics management information systems (LMIS) presents an opportunity to estimate health impact.

**Methods:**

The impact of LMIS on reductions in stockouts was estimated from primary data and peer-reviewed literature, with three scenarios of impact: 5% stockout reduction (conservative), 10% stockout reduction (base), and 15% stockout reduction (optimistic). Stockout reduction data was inverted to stock availability and improved coverage for vaccines and essential medicines using a 1:1 conversion factor. The Lives Saved Tool (LiST) model was used to estimate health impact from lives saved in newborns and children in Mozambique, Tanzania, and Ethiopia between 2022 and 2026 across the three scenarios.

**Results:**

Improving coverage of vaccines with a digital LMIS intervention in the base scenario (conservative, optimistic) could prevent 4,924 (2,578–6,094), 3,998 (1,621–4,915), and 17,648 (12,656–22,776) deaths in Mozambique, Tanzania, and Ethiopia, respectively over the forecast timeframe. In addition, scaling up coverage of non-vaccine medications could prevent 17,044 (8,561–25,392), 21,772 (10,976–32,401), and 34,981 (17,543–52,194) deaths in Mozambique, Tanzania, and Ethiopia, respectively. In the base model scenario, the maximum percent reduction in deaths across all geographies was 1.6% for vaccines and 4.1% for non-vaccine medications.

**Interpretation:**

This study projects that digitalization of last-mile LMIS would reduce child mortality by improving coverage of lifesaving health commodities. This analysis helps to build the evidence base around the benefits of deploying digital solutions to address health challenges. Findings should be interpreted carefully as stockout reduction estimates are derived from a small number of studies.

## Introduction

Digital health has become a widely recognized approach to addressing a range of health needs, including efforts to advance universal health coverage and achieve the ambitious Sustainable Development Goals. A growing number of normative agencies and partners are developing frameworks and methodologies to standardize economic evaluation and impact measures from digital health interventions, but at present there is limited quantitative evidence on the potential impact of digital interventions on health outcomes [1]. Measuring the value of digital in health can be challenging due to many positive and negative externalities (e.g. workforce motivation, data privacy violations), the wide variety of potential outcome measures (e.g. health impact, financial savings) and many different methods for measuring this impact [2].

A limited but growing body of peer-reviewed evidence on the impact of digital health interventions on improved care delivery and coverage of health services and commodities presents an opportunity to estimate health impact [3–7]. These studies measure a variety of outcomes, from increased adherence to medical schedules to reductions in stockouts to changes in behavior. Stockouts are generally defined as a commodity that is expected to be provided at a health facility but that has zero reported stock at any point during a defined period. Some of these outcomes can be put into the Lives Saved Tool (LiST), a modelling program in the Spectrum software package that analyses how increases in the coverage of specific health interventions prevent additional deaths and affect mortality rates over time. LiST uses empirical evidence on the effectiveness of health interventions and proportion of deaths that can be averted by that intervention, referred to as the affected fraction [8]. LiST is validated by comparing measured mortality rates in low- and middle-income countries (LMICs) to mortality rates in calculated in LiST. More than 100 peer-reviewed publications have used LiST for program evaluation, strategic planning, and advocacy [9].

**Fig 1:**
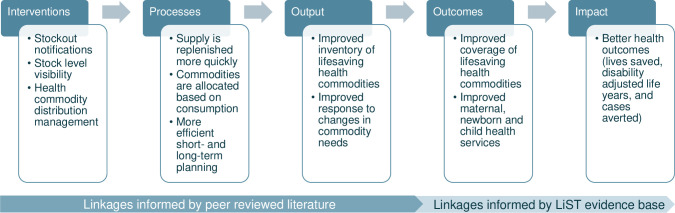
Impact model connecting digital health and improved health outcomes through LMIS. Abbreviations: LiST, Lives Saved Tool.

The purpose of this research is to contribute to the evidence base linking digital health interventions, health system outcomes, and health impact estimates. This study specifically focuses on the estimated health impact from digitalizing last-mile electronic logistics management information systems (LMIS) with inventory management, stock level notification, and distribution functionality. In LMICs, paper-based systems requiring manual data entry of health information are common at the lowest levels of the health system. Digitalized last-mile LMIS can improve the supply and distribution of health commodities by automating the various steps in the supply chain thus reducing stockouts, waste and supply chain inefficiencies [10]. Fig 1 demonstrates the evidence-informed links between digitized supply chain interventions and health impact [11].

Mozambique, Tanzania, and Ethiopia were selected as the geographies of interest given that they are all in sub-Saharan Africa, where some of the largest health disparities exist relative to other geographies. In addition, these countries are in different digital health maturity levels. Digital health market maturity impacts the pace and ability of digital health innovations to scale in a given geographic context. The maturity level for a given country was determined using a model that contains indicators for digital political and regulatory factors, digital literacy, and digital infrastructure [12]. Each of these countries have digitalized LMIS systems that were implemented sub-nationally between 2013–2014. However, last-mile paper-based systems remain standard where these systems have not yet reached or where the government retains the paper-based system in parallel as a backup to the digital system. Lastly, these geographies also represent a cross section of LMICs with different population sizes, mortality rates, disease burdens, and baseline coverage rates for the interventions of interest in the analysis.

This research contributes to the global body of evidence on the health impact of digitalizing last-mile LMIS and proposes a replicable method for digital health researchers to connect peer-reviewed literature on the impact of digital health interventions with the LiST model or other modelling tools.

## Materials and methods

This study estimated national changes in newborn and child health interventions and associated health impact based on the implementation of a digitalized last-mile LMIS between the years 2022 and 2026 in Mozambique, Tanzania, and Ethiopia. Based on available evidence, the implementation of the digitalized last-mile LMIS is projected to result in improvements in coverage of select lifesaving commodities. The study calculated three scenarios (conservative, base, and optimistic) with different coverage improvement rates for each country and compared these to the status quo where there is no change in coverage for the medicines and vaccines of focus in this analysis.

### Developing scenarios for stock availability increases

We conducted a literature review to identify secondary data sources and augmented this with key informant interviews with implementing partners to identify changes in intervention coverage of lifesaving commodities based on the implementation of a digitalized last-mile LMIS. We used source material from a variety of key documents to inform the research and data collection for this study: World Health Organization (WHO) guideline recommendations on digital interventions for health system strengthening [1], GSMA Scaling Digital Health in Developing Markets [13], WHO Digital Implementation Investment Guide [14], and PubMed and Google Scholar searches. The literature review was restricted to observational studies, randomized control trials, meta-analyses, and systematic reviews and included the search terms "digital technolog*" OR "mobile application" OR "digital tool*" AND "cost effective*" OR "health impact*" OR "impact eval*" OR "technology assess*" OR "comparative effectiveness research" OR "cost-benefit analysis" OR "treatment outcome." The search method identified 136 articles. These articles were further filtered to exclude articles published prior to 2010 given that digitized last -mile LMIS systems were not largely available in these geographies at this time and exclude those that exclusively focused on high-income geographies. Of the remaining 44 articles, a subset of 6 articles was selected due to their focus on digital, last-mile LMIS interventions. Of the final 6 articles, 3 articles addressed digital, last-mile LMIS interventions with measurable impact on stockout rates and/or vaccine availability (S1 Fig) [15–17].

The data gathered in the literature review informed the range of the expected magnitude of change in the coverage of an intervention (e.g. vaccines or non-vaccine essential medicines) that would be expected to improve due to digital health (Table 1). Based on the data, the expected range of magnitude of change from a reduction in stockouts was 5 to 14 percentage points. This range was validated with additional primary data collected from implementing partners supporting deployments of LMIS specifically in Mozambique. Two sources from digitalized last-mile LMIS implementations in Tanzania showed a 5 percentage point decrease in stockout rates and 13 percentage point decrease in stockout rates. A study of a digital, last-mile LMIS implementation in India showed an increase in stock availability of 8 percentage points. Primary research data from a OpenLMIS implementation in Mozambique indicated a reduction in stockout rates of 14 percentage points in public health medicines one year after deployment of the intervention (unpublished primary data provided by personal correspondence on February 10, 2021). OpenLMIS is an open-source, cloud-based digitized LMIS.

**Table 1:** Summary of the impact of digitalized, last-mile Logistics Management Information Systems (LMIS) on commodity stock levels.

Source	Geography	Impact	Timeline of measured change	Absolute magnitude of change	Definition of impact
Peer-reviewed literature	Tanzania (National, deployed only at central level)	Medicine stockout rates	12 months	Decreased by 13%	Measured as the existence of any zero-stock event in one or multiple sessions in a vaccine-facility-month [15]
Peer-reviewed literature	Tanzania (3 regions)	Vaccine stockout rates	12 months	Decreased by 5%	Measured as the existence of any zero-stock event in one or multiple sessions in a vaccine-facility-month [16]
Peer-reviewed literature	India (2 districts in state of Uttar Pradesh)	Vaccine availability	13 months	Increased by 8%	Measured as the percentage of vaccines available at the start of session day on which the evaluation took place [17]
Primary data	Mozambique (4 provinces)	Medicine stockout rates	12 months	Decreased by 14%	Rates measured one year after deployment of LMIS

Base (10%), optimistic (15%), and conservative (5%) coverage scenarios enabled the model to capture the range in absolutely effect size across studies identified in Table 1. Each scenario was compared to a status quo (no changes in coverage) and assumed a 1:1 conversion between increased availability of a medicine or vaccine and increased use of that medicine or vaccine.

### Other LiST model inputs

For baseline mortality rates, health status, and effectiveness of interventions, the model used LiST-generated data from peer-reviewed journals, the United Nations Population Division, the Demographic and Health Surveys Program, and the Multiple Indicator Cluster Surveys [18]. Only the newborn and child health interventions that can be modelled in LiST were included in this analysis. The analysis excluded interventions where baseline coverage data were not available or estimated to be zero in LiST. It also excluded interventions that were not linked to a medicine or vaccine included in an LMIS.

### Data analysis

**Fig 2:**
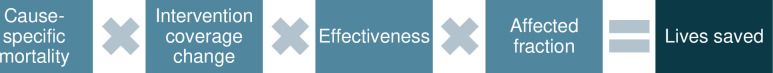
Lives Saved Tool (LiST) impact calculation.

The calculation for modelling lives saved from changes in intervention coverage in LiST is shown in Fig 2 [11]. Cause-specific mortality is calculated by multiplying number of births by overall mortality rates by the proportion of deaths estimated as being due to specific causes. Mortality rates are specific to each disease area based on the UN Inter-agency Group for Child Mortality Estimation and include estimates for children (1–59 months of age) based on deaths per 1,000 live births. Effectiveness is defined as the proportion of pathogen- or cause-specific deaths averted by a specific intervention. The LiST model eliminates potential double counting when scaling multiple interventions at once by using cause-specific mortality and applying additional interventions to the remaining residual deaths [18]. The model assumes that each death is due to a single cause and that each death can only be prevented once.

The analysis compared the three scenarios of impact to a counterfactual of the status quo or no change in coverage for the medicines and vaccines of focus, from 2022 to 2026. Given that the literature review and key informant interviews revealed rapid (12-month) rollout processes for the digitalized last-mile LMIS, the model assumed a one-year timeline for scale-up to final coverage rate increases in base, optimistic, and conservative scenarios, and then held constant for the remainder of the forecast timeframe.

In addition to variation in digital health maturity, each country varied in baseline population, health status, and coverage of interventions applicable for this analysis as shown in Table 2. When a baseline coverage percent was too high to model to the full effect size of a scenario, the coverage was increased to 99%.

**Table 2:** Country-specific baseline coverage (%) in Lives Saved Tool (LiST) software.

*Medical commodity*, *or intervention*		Mozambique [19]	Tanzania [20]	Ethiopia [21]
**Vaccines**	BCG	94***·***0	91***·***0	69***·***0
	DPT	88***·***0	89***·***0	69***·***0
	Hepatitis B	88***·***0	89***·***0	68***·***0
	HiB	88***·***0	89***·***0	68***·***0
	Measles	87***·***0	88***·***0	58***·***0
	Pneumococcal	80***·***0	83***·***0	63***·***0
	Polio	88***·***0	89***·***0	72***·***0
	Rotavirus	88***·***0	85***·***0	68***·***0
	Tetanus toxoid	86***·***0	91***·***0	93***·***0
**Non-vaccines**	Antibiotics for preterm or prolonged PROM	48***·***5	32·2	19***·***6
	Antibiotic treatment for dysentery	31***·***6	2·8	9***·***3
	Injectable antibiotics	64***·***8	62·6	26***·***2
	Oral antibiotics for pneumonia	59***·***4	55·4	29***·***4
	Oral rehydration solution	45***·***9	44·8	29***·***5
	Parenteral administration of antibiotics	48***·***5	32·2	19***·***6
	Syphilis detection and treatment	22***·***4	56·2	15***·***7
	Vitamin A treatment of measles	64***·***0	99·0	48***·***0
	Zinc treatment of diarrhea	30***·***9	17·5	33***·***3

Abbreviations: BCG, Bacillus Calmette–Guérin; DPT, diphtheria-pertussis-tetanus; HiB, Haemophilus influenzae type B; PROM, premature rupture of membrane.

## Results

**Fig 3:**
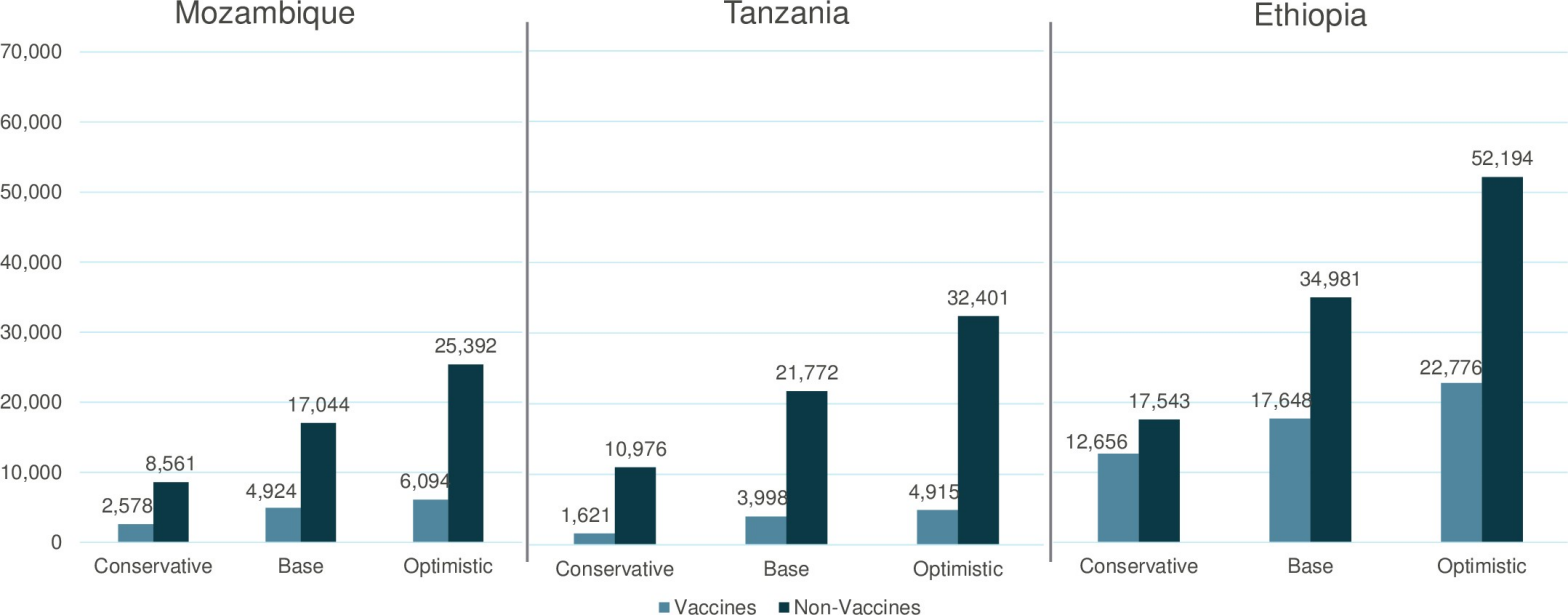
Total lives saved for newborns and children 0–59 months over five years when scaling up coverage of commodities across Mozambique, Tanzania, and Ethiopia.

Scaling up coverage of vaccines over five years with a base scenario of 10% reduction in stockouts (conservative scenario of 5% reduction in stockouts–optimistic scenario of 15% reduction in stockouts) could prevent approximately 4,924 (2,578–6,094), 3,988 (1,621–4,915), and 17,648 (12,656–22,776) deaths in newborns and children under five in Mozambique, Tanzania, and Ethiopia, respectively. Scaling up coverage of non-vaccine medications accounts for a greater share of the total lives saved impact and could prevent approximately 17,044 (8,561–25,392), 21,772 (10,976–32,401), and 34,981 (17,543–52,194) deaths in newborns and children under five in Mozambique, Tanzania, and Ethiopia, respectively (Fig 3). Vaccine and non-vaccine essential medicine interventions had different effect sizes on mortality in newborns and children under five, with non-vaccine medicines accounting for larger reductions in mortality (Table 3).

**Table 3:** Mortality reductions in children under five (%)^a^.

	Mozambique	Tanzania	Ethiopia
**Scenario 1: Conservative**			
** • Vaccine**	0***·***5	0***·***5	0***·***9
** • Non-vaccine**	2***·***0	2***·***0	1***·***8
**Scenarios 2: Base**			
** • Vaccine**	1***·***1	0***·***9	1***·***6
** • Non-vaccine**	3***·***9	4***·***1	3***·***6
**Scenario 3: Optimistic**			
** • Vaccine**	1***·***3	1***·***1	2***·***1
** • Non-vaccine**	5***·***9	6***·***0	5***·***4

^a^ Mortality reductions are based on the percent change in total number of deaths without any intervention compared to the total number of deaths with the intervention. The expected number of under-five child deaths without the intervention is estimated to be 423,713 in Mozambique, 530,262 in Tanzania, and 960,063 in Ethiopia over five years.

The non-vaccine interventions with the largest impact on under-five newborn and child mortality across all three geographies were treatment of neonatal sepsis and pneumonia and oral antibiotics for pneumonia (S2 Fig). Across all three countries, these interventions showed the greatest impact on newborn and child mortality when scaled with all lifesaving commodities. In Mozambique, scaling up coverage of neonatal sepsis and pneumonia treatment showed the greatest impact and was estimated to save 5,269 (2,649–7,836) lives of newborns and children under five over five years. In Tanzania, scaling oral antibiotics for pneumonia showed the greatest impact with an estimated 6,818 (3,392–10,247) lives saved in newborns and children under five. Similar to Mozambique, scaling up coverage of neonatal sepsis and pneumonia treatment showed the greatest impact in Ethiopia with an estimated 10,700 (5,364–15,965) lives saved over five years. Scaling up oral antibiotics for pneumonia showed the second highest impact in terms of lives saved in newborns and children under five in Mozambique 5,220 (2,604–7,839) and Ethiopia 10,181 (5,069–15,297).

The health impact from scaling up vaccine coverage was much less than that from scaling up non-vaccine medicines (Fig 3). Scaling up pneumococcal vaccine coverage showed the greatest impact in Mozambique and Ethiopia, with the number of lives saved for newborns and children under five estimated to be 1,485 (766–2,159) and 4,427 (3,011–6,123), respectively over five years (S3 Fig). In Tanzania scaling up diphtheria-pertussis-tetanus (DPT) vaccine showed the highest number of lives saved followed by the pneumococcal vaccine. The lower health impact from scaling up vaccines in these countries is likely in large part due to the higher baseline coverage rate of vaccines relative to non-vaccine medicines (Table 2). For several vaccines, the coverage could not be increased to the full value given the higher coverage rate.

## Discussion

Implementing a digital health intervention with inventory management, stock level notification, and distribution functionality has the potential to reduce under-five child mortality by improving coverage of lifesaving health commodities. This finding was consistent across commodities and the geographies of Mozambique, Tanzania, and Ethiopia. However, there are many factors that contribute to the impact of digital health interventions. First, the underlying population and health status impacted the total potential effect size of improving stock availability: the healthier the population, the less change was possible due to improving access. In the countries included in this research, the analysis showed that increasing coverage of pneumococcal and DPT vaccines and commodities for sepsis, pneumonia, and diarrhea treatment have the greatest impact on health outcomes, in part because these interventions tackled some of the highest burdens of disease for these geographies. In addition, there are effective health interventions to prevent and/or manage these diseases, resulting in improvements in health outcomes. Second, geographies and commodities that had a high level of baseline coverage also had reduced potential effect size in the model. This is most telling when comparing results between non-vaccine medications and vaccines, with the former having consistently higher impact partly due to the consistently lower baseline coverage levels.

There are several limitations to this work, the first being that the key inputs in the modelling are based on available published literature on the estimated impact of LMIS on improvements in commodity stock levels. The study leverages four studies with published data directly addressing changes in essential medicine and vaccine stock levels following implementation of a digitalized LMIS. These studies were limited to retrospective pre–post designs which do not control for other elements that could be occurring while the intervention is being implemented.

A second important limitation is the assumption that reducing stockouts improves medical commodity coverage using a 1:1 conversion given there are many determinants for increasing coverage of lifesaving medical commodities. On the one hand this 1:1 relationship may be an overestimation because system and governance factors may also have an indirect influence on whether a patient receives a needed health commodity. For example, the infrastructure needed to support supply chains (such as roads, transportation, storage, and electricity) have been shown to influence the level of commodity stockouts in LMICs [22]. In addition, the deployment of a qualified workforce has also been shown to be a factor in ensuring adequate coverage of medical commodities. In Mozambique, the number of doctors and nurses at a health facility impacted the degree of stockouts in those facilities: each additional doctor or nurse per 10,000 visits was associated with 25% lower stockout rate [23]. Furthermore, factors that affect whether a commodity is used (assuming availability of inventory at the point of care) often include people-level influences such as patient care-seeking behavior, or patient ability to pay [24, 25]. While our analysis does not account for the impact of these factors, we acknowledge that they may influence the degree to which a digital health intervention could improve service coverage, increase access to health commodities, and ultimately reduce mortality.

On the other hand, the model results may be an underestimation because reducing stockouts can increase trust in the health system, leading to a higher volume of facility visits and an increase in demand [26, 27]. These factors suggest that the magnitude of change for a digital health intervention may be confounded by other changes or interactions in the health care system. Scenario analysis was performed to capture overall uncertainty in the analysis and provide ranged estimates. It should also be noted that certain interventions cannot be modelled in LiST, and thus the analysis does not estimate the potential full impact of implementing an LMIS.

This analysis builds on the limited evidence base for the impact and social return for digital health in LMICs. Without data on health impact, stakeholders and decision-makers lack the necessary information to guide investments and efforts to improve coverage of lifesaving commodities. When it comes to evidence for the impact of digital health interventions, more published research, including data collection and making data available in the public domain, should be a priority moving forward. The promising findings of this analysis highlight the need to expand digital health impact research for additional digital health interventions to inform investment decisions. One important next step will be to link impact analyses, like this, with costing information related to the implementation of relevant digital health interventions to develop a social return on investment estimation. For some digital health interventions listed in the WHO digital health classification, different types of impact measurements such as cost savings or time savings may be more appropriate to capture the value of different digital solutions. In addition to expanding the body of evidence for impact, understanding the linkages of countries’ digital health maturity and the magnitude of potential impact is an important next step for guiding investment decisions.

## Supporting information

S1 Fig Literature review and secondary data selection process. Abbreviations: LMIS, logistics management information systems.
(TIF)

S2 Fig Total lives saved in newborns and children under five from non-vaccine medicines coverage scale up over 5 years (base scenario). *In Tanzania, Vitamin A for measles coverage is already at 99%. Consequently, no additional lives saved could be modelled due to increase in coverage for this intervention. Abbreviations: ORS, oral rehydration solution; PROM, premature rupture of membrane; Trt, treatment.
(TIF)

S3 Fig Total lives saved in newborns and children under five from vaccine coverage scale up over five years (base scenario). Abbreviations: DPT, diphtheria-pertussis-tetanus; HiB, Haemophilus influenzae type B.
(TIF)

S1 Dataset Modeled intervention coverage and lives saved in LiST for newborns and children under five in Ethiopia, Tanzania, and Mozambique. Abbreviations: AIM, AIDS Impact Model; ART, antiretroviral therapy; BCG, Bacillus Calmette- Guérin; DemProj, Demography model; DHS, Demographic and Health Surveys; DPT, diphtheria-pertussis-tetanus; FamPlan, Family Planning; HiB, Haemophilus influenzae type B; HIV, human immunodeficiency virus; LiST, Lives Saved Tool; ORS, oral rehydration solution; PMTCT, Prevention of Mother-to-Child Transmission of HIV; PROM, premature rupture of membrane; UNAIDS, Joint United Nations Programme on HIV/AIDS; WPP, United Nations World Population Prospects.
(XLSX)
