# Supplementary figures and images for: Estimation of health impact from digitalizing last-mile Logistics Management Information Systems (LMIS) in Ethiopia, Tanzania, and Mozambique: A Lives Saved Tool (LiST) model analysis
Source: PLoS One. 2021 Oct 25;16(10):e0258354. doi: 10.1371/journal.pone.0258354 (PMC8544866; doi:10.1371/journal.pone.0258354)

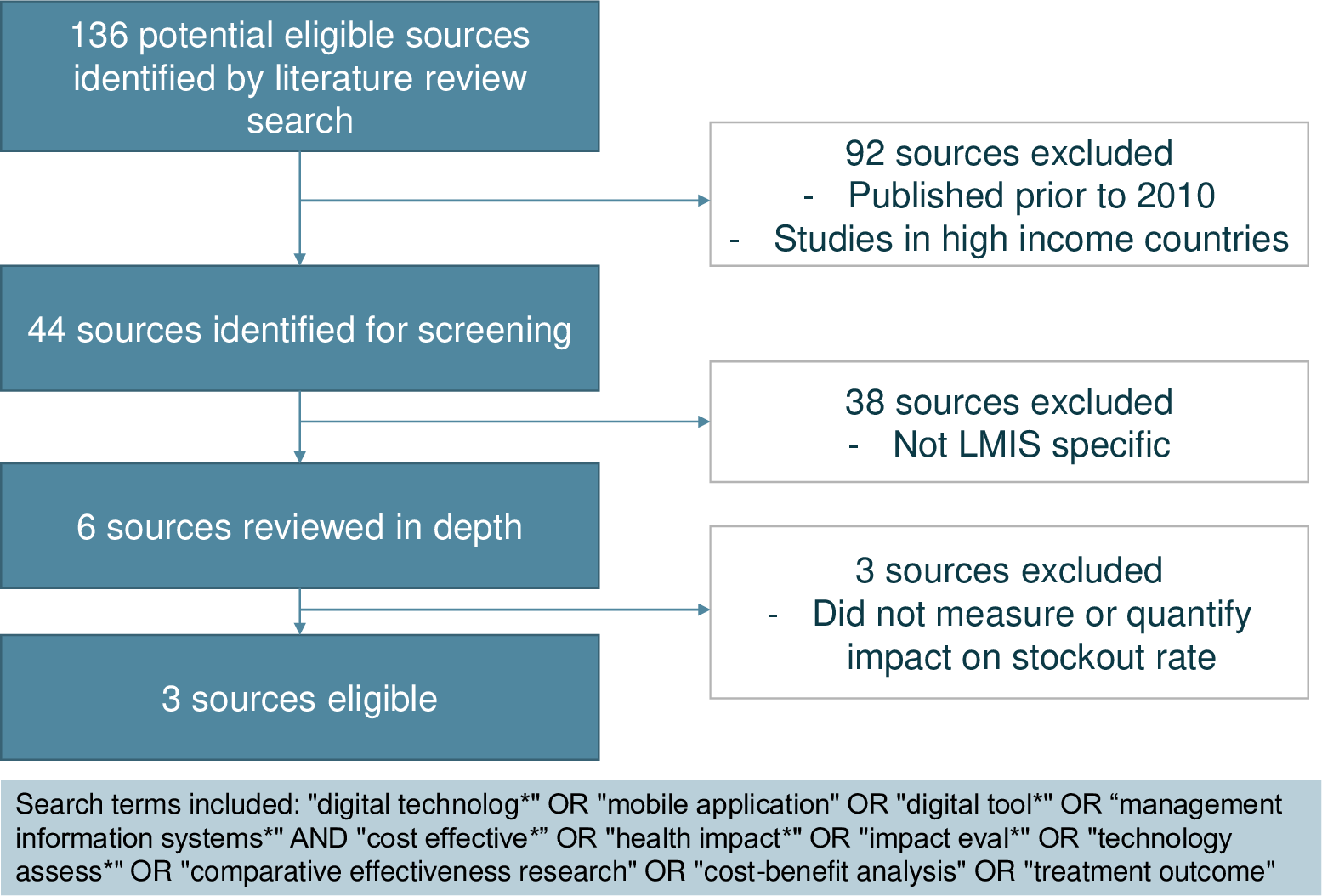

Supplement: S1 Fig — Abbreviations: LMIS, logistics management information systems. (TIF) [file pone.0258354.s001.tif]

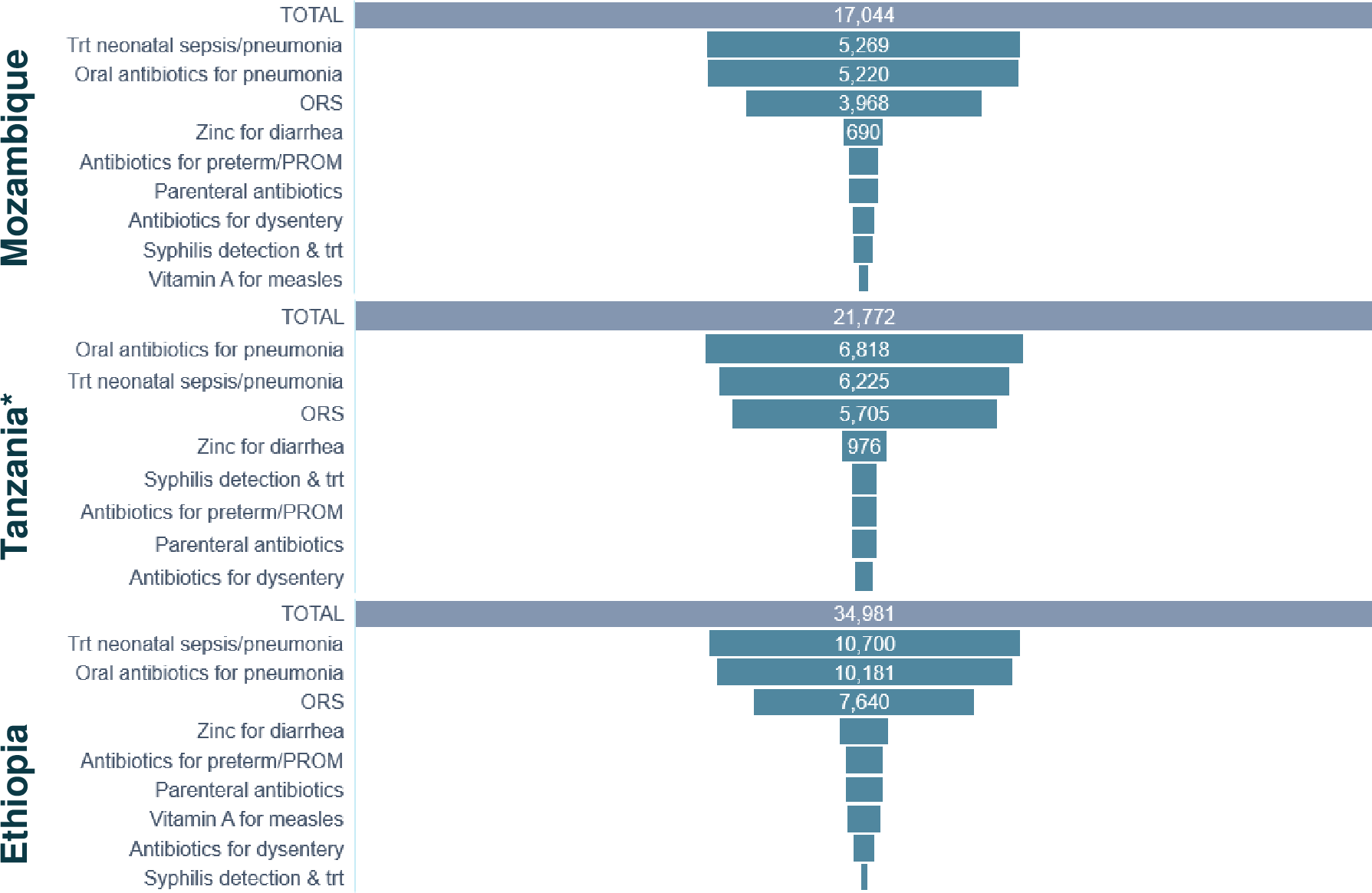

Supplement: S2 Fig — *In Tanzania, Vitamin A for measles coverage is already at 99%. Consequently, no additional lives saved could be modelled due to increase in coverage for this intervention. Abbreviations: ORS, oral rehydration solution; PROM, premature rupture of membrane; Trt, treatment. (TIF) [file pone.0258354.s002.tif]

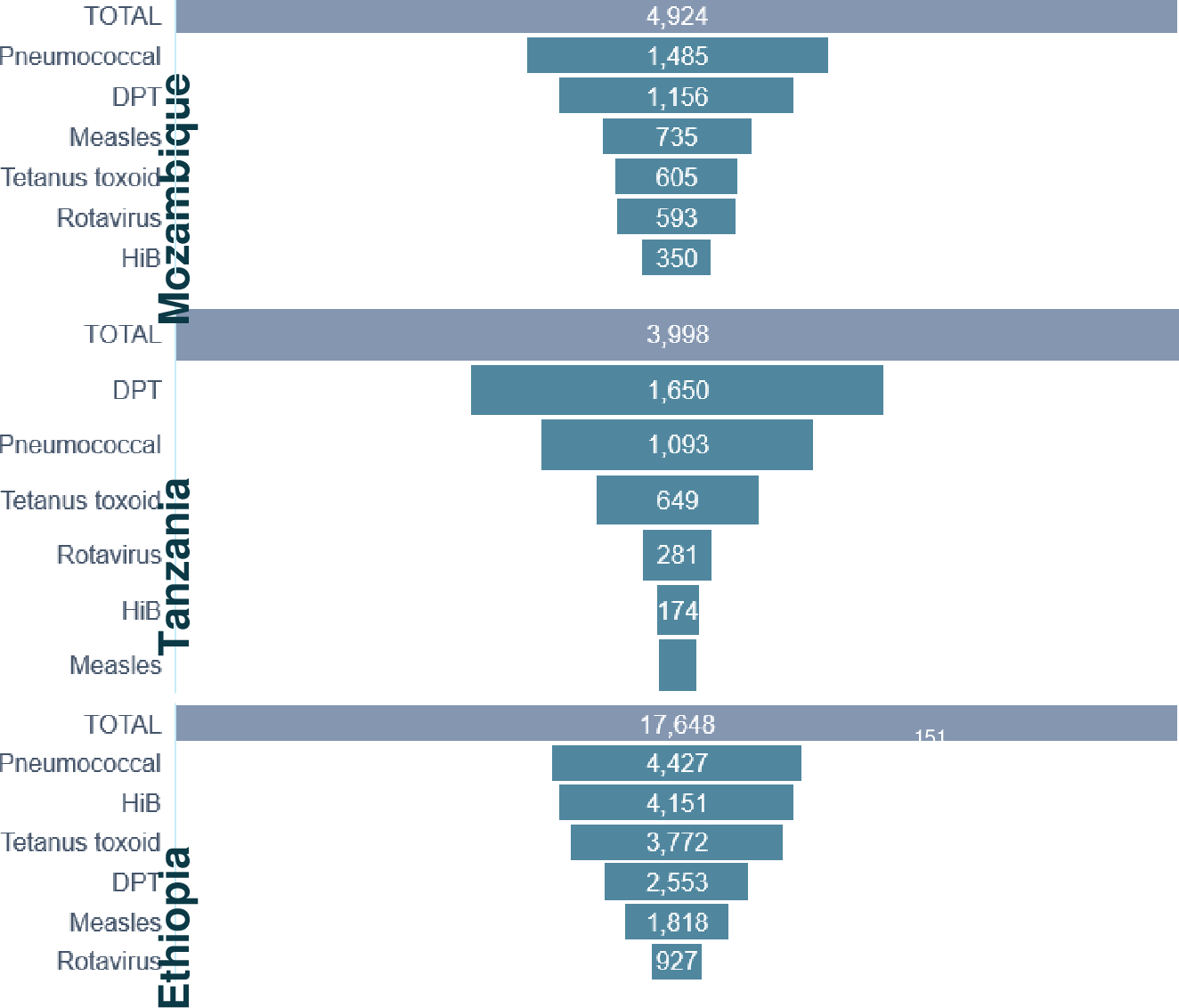

Supplement: S3 Fig — Abbreviations: DPT, diphtheria-pertussis-tetanus; HiB, Haemophilus influenzae type B. (TIF) [file pone.0258354.s003.tif]
